# The Evolving Landscape of Immune Regulation and Immunotherapy in Cholangiocarcinoma and Biliary Tract Cancer

**DOI:** 10.3390/cancers18122001

**Published:** 2026-06-20

**Authors:** Emanuelle Rizk, Patrick Foley, Soravis Osataphan

**Affiliations:** Beth Israel Deaconess Medical Center, Harvard Medical School, Boston, MA 02215, USA

**Keywords:** cholangiocarcinoma, immunotherapy, immune checkpoint inhibitors, tumor microenvironment, PD-1/PD-L1, combination therapy, biomarkers, adoptive cell therapy

## Abstract

Cholangiocarcinoma (CCA) is an aggressive and molecularly heterogeneous malignancy characterized by a profoundly immunosuppressive tumor microenvironment (TME) and historically limited therapeutic options. Recent advances have redefined the treatment paradigm, with phase III trials establishing chemoimmunotherapy as a standard of care and multi-omic profiling elucidating the interplay between tumor genomics, stromal architecture, and immune regulation. Despite these gains, durable clinical benefit remains confined to a minority of patients, reflecting convergent mechanisms of primary and acquired resistance—including immune exclusion, myeloid-dominant suppression, and genotype-driven “cold” tumor states. In this review, we synthesize emerging insights into the immune landscape of CCA, integrating data from single-cell, spatial, and translational studies to define the cellular and molecular circuits governing immune evasion. Beyond canonical biomarkers such as mismatch repair and microsatellite status, we highlight how spatial organization of immunity—in particular, tertiary lymphoid structures, dynamic myeloid and stromal interactions, and pathway-level features—shape immunotherapy responsiveness. We also examine how tumor-intrinsic alterations, including IDH1 mutation, FGFR2 fusions, KRAS activation, and MTAP loss, define distinct immunologic phenotypes with direct implications for immunotherapeutic response and biomarker-driven patient selection. We evaluate the expanding clinical trial landscape of immunotherapy in CCA and more broadly in BTC, including adoptive cell therapies and cancer vaccines. Together, these advances position CCA as a paradigm of how tumor genotype and microenvironment co-evolve to define immunotherapy sensitivity and resistance.

## Simple Summary

Cholangiocarcinoma (CCA) is an aggressive malignancy defined by a profoundly immunosuppressive tumor microenvironment (TME) and substantial molecular heterogeneity. Chemoimmunotherapy has recently been established as standard of care in the first line setting, but durable clinical benefit remains confined to a minority of patients. In this review, we examine the cellular and molecular underpinnings of immunotherapy response in CCA, integrating tumor-intrinsic genomic features, TME architecture, and emerging therapeutic strategies including adoptive cell therapies and cancer vaccines, thus positioning CCA as a paradigm for understanding the interactions between tumor genotype, microenvironment, and immunotherapy sensitivity.

## 1. Introduction

Cholangiocarcinoma (CCA) represents a diverse group of aggressive epithelial malignancies originating from the biliary tree or hepatic progenitor cells, accounting for approximately 3% of all gastrointestinal cancers and representing the second most common primary hepatic malignancy after hepatocellular carcinoma [1]. The global incidence of CCA has been steadily increasing over the past several decades, with significant geographic variation reflecting differences in risk factors, including liver fluke infections in Southeast Asia, primary sclerosing cholangitis in Western countries, and hepatitis B and C infections worldwide [2,3,4,5].

CCA is anatomically classified into intrahepatic cholangiocarcinoma (iCCA), perihilar cholangiocarcinoma (pCCA), and distal cholangiocarcinoma (dCCA), each presenting distinct molecular profiles, tumor microenvironment, clinical behaviors, and therapeutic challenges [6,7]. The majority of patients present with advanced, unresectable disease at diagnosis, contributing to the dismal overall survival rates, with a 5-year survival of less than 10% for advanced-stage disease [8]. Previous standard first-line systemic therapy has relied on cytotoxic chemotherapy, with gemcitabine plus cisplatin providing only modest benefit and yielding a median overall survival of approximately 11 to 12 months [9,10]. Immune checkpoint inhibitors with PD-L1 in addition to chemotherapy has been shown to improve the outcomes in patients in the two phase III international clinical trials [11,12]. However, the improvement is modest with 10–20% of patients with prolonged response to therapy [13,14]. This is likely, in part, driven by the complex and heterogeneous immunological landscape of CCA characterized by an immunosuppressive tumor microenvironment (TME) that presents significant barriers to effective immune-mediated tumor clearance [6]. Understanding the intricate interplay between tumor cells, immune cells, and stromal components within the CCA microenvironment is crucial for understanding resistance mechanisms and developing rational immunotherapeutic strategies and combination therapies.

This review provides a comprehensive overview of immune regulation in CCA, examines current clinical evidence for immunotherapy, discusses emerging therapeutic approaches, and identifies future research directions aimed at improving outcomes for patients with this challenging malignancy.

## 2. The Immunological Landscape of Cholangiocarcinoma

### 2.1. Tumor Microenvironment Subtype Classification

The tumor microenvironment (TME) of CCA is profoundly heterogenous, and its systematic characterization has emerged as a central framework for understanding disease biology and prognosis. The most comprehensive TME-based classification to date was established in a cohort of 511 iCCA where unsupervised clustering was performed on immune and stromal metagene signatures derived from computational cellular deconvolution (MCP-counter), a deliberate methodological choice that prioritizes microenvironmental architecture over tumor-intrinsic transcriptional noise [15]. This approach resolved four reproducible subtypes: immune-desert (~45%), immunogenic (~10%), myeloid-enriched (~20%), and mesenchymal-like (~25%), the latter defined by fibroblast and hepatic stellate cell enrichment [15]. Survival tracked predictably with immune activation: the immunogenic subtype carried the most favorable prognosis, while the mesenchymal subtype conferred the worst survival [15].

A critical question raised by these population-level classifications is whether immune contexture is stable within individual tumors or merely reflects averaged tissue composition. A multi-omic study of 16 patients with multifocal iCCA—integrating whole-exome sequencing, bulk and single-cell RNA sequencing, methylation profiling, and multiplexed immunostaining across 66 tumor foci—found that immunophenotypes remained consistent within individual patients despite substantial genomic, transcriptional, and epigenomic divergence across foci [16]. This patient-level immunophenotypic coherence has direct implications for biopsy strategy and treatment selection in the multifocal setting. Complementary spatial resolution was provided by a multi-region study applying RNA-seq and multiplexed immunofluorescence across 207 subregions from 45 iCCA patients, which classified tumors into sparsely infiltrated (~27%), heterogeneously infiltrated (~58%), and highly infiltrated (~16%) subgroups—each with distinct immunogenomic features and immune evasion mechanisms [17]. Notably, over half of iCCAs displayed intratumoral heterogeneity of immune infiltration, underscoring the limitations of single-region sampling and urgent need for a spatially informed biomarker strategy [17].

In broader transcriptomic classifications that incorporate tumor cell intrinsic features, mesenchymal and immune modules emerge consistently and correlate with survival outcomes, though their relative proportions are attenuated, as tumor-intrinsic characteristics dominate these classifications [18,19,20,21]. In one study, an unsupervised clustering analysis of the global tumor transcriptome in extrahepatic CCA (n = 189)—performed without prior immune or stromal deconvolution—identified four classes: metabolic (~20%), proliferative (~20%), mesenchymal (~50%), and immune (~10%) [20]. Proteomic clustering of 221 intrahepatic CCA samples from China similarly confirmed that mesenchymal- and immune-related protein signatures define distinct subgroups, with the mesenchymal subtype associated with worse survival [22,23]. Complementary evidence from 454 patients in a real-world cohort of all three BTC subtypes using mutation-based clustering similarly resolved four genomically defined clusters with distinct immune biomarker profiles, reinforcing the concept that molecular drivers—rather than anatomical subtype alone—are key determinants of immune contexture in BTC [24].

### 2.2. Drivers of Intertumoral Heterogeneity

Several intersecting factors underlie intertumoral TME heterogeneity in CCA, including disease etiology, carcinogenic exposure history, and tumor genotype. These influences are not merely coincidental—they appear to shape distinct epigenomic programs that in turn determine immune contexture.

Liver fluke infection (*Opisthorchis viverrini* and *Clonorchis sinensis*) is the most extensively characterized etiological driver of TME divergence. Fluke-associated CCA harbors a distinct mutational and epigenetic architecture, characterized by promoter CpG island hypermethylation, elevated APOBEC mutational burden, enrichment of *ARID1A*, *BRCA1/2*, and *TP53* mutations, and widespread gene expression silencing—including marked downregulation of immune-related genes and reduced immune cell infiltration [25,26]. Integrative enhancer profiling has further mechanistically linked fluke-positive CCA to aberrant activation of estrogen signaling, with fluke-positive tumors forming a coherent epigenomic cluster (ESTRO) distinct from immunogenic and oxidative phosphorylation-enriched subtypes—and demonstrating the lowest immune cell infiltration of the three groups, potentially via TNF-mediated immune evasion [26]. Importantly, the immunosuppressive landscape of fluke-positive CCA may not be driven solely by immune exclusion but rather by a carcinogen-induced epigenetic remodeling that fundamentally reprograms enhancer activity and downstream immune signaling.

In contrast to these immune-cold etiologies, PSC-associated CCA displays a more heterogeneous immune landscape, encompassing both inflamed and immune-cold phenotypes [27]. The pathophysiology underlying this diversity remains incompletely understood, though gut dysbiosis-mediated hepatic immunosuppression—operating through TLR4-dependent CXCL1 induction and subsequent CXCR2+ polymorphonuclear MDSC accumulation—has been identified as one potential mechanism linking PSC-related microbiome alterations to a protumorigenic hepatic immune environment [28].

IDH1-mutant CCA is a biologically distinct subtype with an indolent clinical course and eligibility for targeted therapy but is characterized by low infiltration of cytotoxic immune effectors including T cells, B cells, and NK cells and lower PD-L1 expression [24,29,30,31]. Mechanistically, the IDH1 neomorphic product 2-hydroxyglutarate (2-HG) suppresses T cell antitumor immunity through multiple complementary pathways including direct impairment of T cell IFN-γ expression through TET2 inactivation and—as recently demonstrated—selective hypermethylation and silencing of *CGAS*, which disables innate immune sensing, suppressing the cGAS-STING pathway, and contributes to the immunologically cold tumor microenvironment [29,32]. In addition, IDH1 mutated CCA produced CCL2 to increase M2-like TAMs [30]. Critically, mIDH1 inhibition with ivosidenib reverses these defects in a coordinated sequence: TET2 reactivation and genome-wide DNA demethylation restore IFN-γ responsiveness in tumor cells; derepression of *CGAS* and transposable element–encoded reverse transcriptase activity generate endogenous dsDNA that activates cGAS-STING via a viral mimicry mechanism, triggering type I interferon signaling and CD8+ T cell recruitment [29,32]. In preclinical models, the antitumor effect of ivosidenib was completely abrogated by CD8+ T cell depletion [29]. Acquired resistance to mIDH1 inhibition is accompanied by the emergence of MAPK pathway mutations, which blunt IFN-γ-driven gene expression, thereby attenuating the immunologic response that underlies ivosidenib’s efficacy [33]. These findings provide strong mechanistic rationale for combining IDH1 inhibition with immune checkpoint blockade, and early-phase clinical data are beginning to test this strategy.

FGFR2 fusions and mutations consistently associate with reduced CD4+, CD8+, and macrophage infiltration, lower TMB, diminished PD-L1 expression, and downregulated immune pathways across transcriptomic, proteomic, and spatial immunophenotyping studies [17,22,24,34,35]. A causal basis for this immune-cold phenotype was established by the finding that the FGFR2::BICC1 fusion directly suppresses CD4+, CD8+, and macrophage infiltration in vivo [22]. Paradoxically, however, the *FGFR2::BICC1* fusion junction itself may generate immunogenic neopeptides capable of priming neoantigen-specific T cell responses, raising the possibility that *FGFR2*-rearranged tumors harbor latent immunogenic potential that is actively suppressed rather than absent [22,36].

In contrast to the immune-cold programs driven by IDH1 and FGFR2 alterations, *KRAS* mutation is associated with a qualitatively distinct immune contexture characterized by elevated myeloid infiltration—particularly macrophage enrichment—upregulation of inflammatory signaling pathways including the MAPK cascade, and enrichment within immune-inflamed transcriptomic subgroups [17,22]. Proteomic subgroup analyses have confirmed that KRAS-mutated iCCA is enriched in a chronic inflammation molecular subtype with high macrophage activation scores [37]. However, the functional consequences for antitumor immunity are complex: KRAS-driven myeloid reprogramming frequently favors immunosuppressive macrophage polarization in other tumor contexts, and the net effect on T cell-mediated antitumor immunity in CCA remains incompletely characterized.

*MTAP* deletion, occurring as part of the recurrent 9p21.3 chromosomal loss that co-deletes *CDKN2A/B*, affects approximately 15% of iCCA and constitutes one of the most frequent genomic alterations in this disease [38]. In iCCA specifically, MTAP loss is consistently associated with a cold immune phenotype: MTAP-loss tumors display significantly lower rates of PD-L1 positivity, lower MSI-high frequency, lower survival, and a trend toward reduced TMB compared to MTAP-intact tumors [38,39]. These iCCA-specific observations are consistent with pan-cancer immunogenomic analyses demonstrating that 9p21 loss—characterized by concomitant deletion of *CDKN2A* and *MTAP*—confers a globally “cold” immune phenotype with reduced abundance of tumor-infiltrating T, B, and NK cells, altered spatial TIL distribution, diminished immune cell trafficking and activation signals, and decreased PD-L1 positivity across multiple solid tumor types [40]. Importantly, the immunosuppressive consequences of 9p21 loss were shown to be driven specifically by *MTAP* loss rather than *CDKN2A* loss [41]. Mechanistically, MTAP loss abolishes enzymatic catabolism of methylthioadenosine (MTA) in the methionine salvage pathway, causing both intracellular and extracellular MTA accumulation within the TME. Extracellular MTA profoundly impairs T cell proliferation, viability, IFN-γ and IL-2 production, and granzyme B expression through two convergent pathways: selective inhibition of PRMT5—an arginine methyltransferase required for cytokine signaling and T cell activation—and agonism of adenosine receptors, which further suppresses effector T cell responses and promotes immunosuppressive macrophage polarization [41,42]. Additionally, MTAP deficiency reprograms tumor-intrinsic cytokine profiles toward a pro-tumoral milieu—suppressing antitumor cytokines including GM-CSF, IL-1α, IL-12, and IFN-γ while enriching immunosuppressive signals—and is associated with tumor cell-intrinsic upregulation of PD-L1, compounding immune exclusion at multiple levels [43]. Together, these mechanisms position MTAP deletion as a metabolic driver of immune exclusion in iCCA.

Collectively, these findings demonstrate that individual driver alterations sculpt mechanistically distinct immune contextures that extend well beyond a simple “hot versus cold” binary—each subtype presenting a unique combination of exclusion mechanisms, checkpoint expression patterns, and latent immunogenic vulnerabilities with direct implications for rational, genotype-informed combination immunotherapy strategies.

### 2.3. Cellular Orchestrators of Immunosuppression and Evasion in Cholangiocarcinoma—Insights from Single-Cell Studies

Single-cell and spatial transcriptomic studies of CCA have converged on a consistent picture of immune exclusion, while revealing important nuances in cellular composition across molecular subtypes. A unifying finding across studies is the dominance of immunosuppressive myeloid cells over cytotoxic lymphocytes within the tumor core. The first comprehensive scRNA-seq atlas of human iCCA and a subsequent spatial multi-omics atlas of 155 iCCA patients both demonstrate that macrophages are the most abundantly infiltrating immune cell type, with T cells largely excluded from the tumor parenchyma and confined to peritumoral margins [44,45]. This spatial pattern was also independently reported through functional ex vivo checkpoint targeting studies [46]. In contrast, multimodal single-cell profiling studies emphasize complementary but distinct suppressive axes operating independently of subtype, with one study identifying hyperactivated MEOX1-driven FOXP3+ Tregs as the dominant lymphoid suppressor of tumor-reactive CD39+CD8+ T cells [47]. and another study demonstrating that TAMs drive PD-L1-mediated immune evasion while their depletion paradoxically triggers compensatory granulocytic MDSC [48]. These single-cell and spatial findings are further supported by translational studies demonstrating that intratumoral CD8+ T cell density independently predicts overall survival and that PD-L1 is expressed in 20–70% of CCA cases with TAMs representing its primary cellular source [48,49]. Complementing these findings, elevated intratumoral FOXP3+ Treg density is independently associated with poor prognosis in iCCA, while a higher CD8+/Treg ratio consistently correlated with improved survival, reflecting the prognostic importance of the balance between effector and suppressive T cell populations [45,47].

### 2.4. Contribution of the Myeloid Compartment

Macrophages are the dominant immune population in the CCA microenvironment, and their contribution was initially framed through the M1/M2 paradigm. In iCCA, alternatively activated (M2) TAMs accumulate preferentially at the tumor margin and promote tumor growth, invasion, and epithelial–mesenchymal transition via STAT3 signaling [50], and both macrophage density and expression of the macrophage growth factor CSF1 (M-CSF) independently predict poorer postoperative survival [51]. This binary framework, however, only partially captures TAM biology: single-cell studies have resolved the compartment into discrete transcriptional states with distinct functions—immunosuppressive C1Q+, CCL18+, and SPP1+ macrophages predominating in more aggressive tumors [52]. The recruitment and polarization of these populations are governed by several chemokine and growth-factor circuits, many demonstrated in CCA. Monocytes are recruited and skewed toward an M2 phenotype through the CCL2–CCR2 axis, with IDH1-mutant tumors and FAP+ CAFs serving as dominant CCL2 sources driving monocyte and MDSC influx [30,53]. CSF1–CSF1R signaling sustains TAM survival and M2 identity, and CSF1R inhibition reduces M2-TAM infiltration and tumor burden in preclinical iCCA models [54]. Large-scale single-cell profiling of primary liver cancer has further delineated five distinct tumor immune microenvironment subtypes—including an immune-suppressive myeloid subtype enriched in ICC and characterized by pro-tumorigenic tumor-associated neutrophil populations that recruit macrophages via CCL4 and suppress CD8+ T cell cytotoxicity through PD-L1, while TAN–TAM co-infiltration activates STAT3 signaling to drive ICC progression and independently predicts poor prognosis [55,56]. MDSC accumulation, driven by tumor-derived GM-CSF and G-CSF, further compounds T cell suppression and represents an additional therapeutic vulnerability [48]. A distinct and underexplored axis in CCA is the macrophage phagocytosis checkpoint: in iCCA, CD47–SIRPα signaling on CD68+ TAMs independently predicts poor prognosis, and CD47 blockade restores macrophage phagocytosis in preclinical models [57].

### 2.5. MAITs

Innate-like lymphocytes represent a complementary axis countering the immunosuppressive CCA microenvironment. Mucosal-associated invariant T (MAIT) cells, an abundant unconventional T cell population in the healthy liver, are depleted from the tumor core in CCA, and their preservation is consistently associated with favorable outcome [58,59]. In a multicenter cohort of 70 patients with iCCA and pCCA, higher density of MAIT cells in tumor-adjacent liver tissue and the presence of intratumoral MAITs were both independently associated with longer survival, and intratumoral MAIT infiltration tracked with a broader antitumor immune signature within the TME [58]. This spatial pattern parallels CD8+ T cell exclusion and highlights MAIT cells as a distinct prognostic dimension beyond conventional T cell contexture. In parallel, NK cell biology offers a therapeutically relevant pathway in the context of frequent HLA class I downregulation [60] and may contribute to immune escape. Elevated MICA expression in iCCA and restoration of NK-cell cytotoxicity via blockade of MICA/B shedding demonstrate that the NK–NKG2D axis remains functionally actionable despite impaired CD8+ recognition [60,61]. Together, these data support incorporating innate-like lymphocytes into TME classification frameworks, which currently focus predominantly on adaptive and myeloid compartments.

### 2.6. T Cells Exhaustion

Beyond the numerical balance of effector and regulatory T cells, the functional state of intratumoral CD8+ T cells—progressive exhaustion marked by co-expression of inhibitory receptors beyond PD-1 such as LAG-3, TIM-3, TIGIT and B7-H3— constitutes a distinct layer of the lymphoid suppressive axis, although the depth of CCA-specific evidence differs markedly across receptors [62]. LAG-3 is the most extensively characterized of these in CCA: PD-1/LAG-3 co-expression marks the exhausted CD8+ T cell pool enriched within TLS-rich tumors and LAG-3 expression by immunohistochemistry correlates with PD-L1 positivity and CD8+ T cell density [63]. TIGIT, which competes with the costimulatory receptor CD226 for the shared ligand CD155 (PVR), is similarly upregulated on dysfunctional CD8+ and NK cells and on Tregs [62]. Single-cell profiling of extrahepatic CCA localizes TIGIT to TOX+ exhausted CD8+ T cells; high TIGIT expression on a tissue microarray independently associates with shorter survival; TIGIT+CD8+ cells exhibit reduced IFN-γ, TNF-α, and TCF-1 with reciprocal upregulation of PD-1 and TIM-3; and anti-TIGIT blockade restores CD8+ effector function and tumor control in patient-derived xenografts [64]. Evidence for the other well-established checkpoint molecules such as TIM-3 (HAVCR2), B7-H3, or VISTA receptors is well established in other tumors but less studied in CCA. TIM-3 (HAVCR2), expressed on exhausted CD8+ T cells, regulatory T cells, and innate immune populations, engages ligands including galectin-9, phosphatidylserine, HMGB1, and CEACAM-1 to attenuate T cell and NK cell effector function, marking a terminally exhausted, frequently PD-1-co-expressing T cell state associated with adverse prognosis and checkpoint-inhibitor resistance across tumor types [65]. B7-H3 (CD276), a B7-family member broadly overexpressed on tumor cells and stroma, suppresses T cell activation while exerting T cell-independent protumoral effects on angiogenesis and tumor metabolism, with high expression correlating with reduced immune infiltration and poor outcome in numerous solid tumors [66]. VISTA (PD-1H), expressed predominantly on myeloid cells and, to a lesser extent, T cells, acts as a negative checkpoint regulator that restrains T cell activation and proliferation and reinforces myeloid-mediated immunosuppression within the tumor microenvironment [67]. Direct study of these receptors in CCA is limited but emerging: in intrahepatic CCA, high tumor-cell TIM-3 expression is present in approximately half of cases, where it correlates with poorer differentiation and with CD4+ and CD8+ tumor-infiltrating lymphocyte density and is associated with shorter disease-free and overall survival [68,69]. B7-H3 has similarly been reported in intrahepatic CCA, where its expression correlates with tumor angiogenesis and adverse clinicopathologic features [70], whereas VISTA remains the least characterized in this setting. Even so, the broader functional roles, prognostic relevance, and therapeutic tractability of these receptors in CCA remain incompletely defined, and further mechanistic and biomarker studies in CCA and BTC will be required to establish whether the immunoregulatory programs characterized in other malignancies fully extend to the biliary tumor microenvironment. Collectively, the co-expression of these receptors defines a functional dimension of the lymphoid axis that compounds Treg- and MDSC-mediated suppression and provides the mechanistic foundation for the combinatorial and next-generation checkpoint strategies discussed later in this review.

### 2.7. B Cells and Tertiary Lymphoid Structures

Notably, B cells and tertiary lymphoid structures (TLSs) represent an important counterweight to this immunosuppressive landscape, though their role in CCA is spatially nuanced. In a large study of iCCA TLS (962 patients across three centers), intratumoral TLS density correlated strongly with favorable prognosis, whereas high peritumoral TLS density was paradoxically associated with worse survival [71]. Multiplex immunohistochemistry revealed different immune compositions, with intratumoral TLS enriched for T follicular helper cells supporting germinal center B cell responses, while peritumoral TLS showed relative Treg enrichment [71]. This opposing spatial prognostic impact was further validated in a separate CCA cohort, where TLS-positive patients showed significantly improved objective response and disease control rates to immunotherapy compared to TLS-negative patients [63]. Within the TLS-rich tumors, there was higher CD8+ T cell exhaustion signatures and PD-1/LAG-3 co-expression—suggesting that TLS may paradoxically prime tumors for checkpoint blockade responsiveness precisely because they foster a pre-exhausted but re-invigorable T cell pool [63].

**Figure 1:**
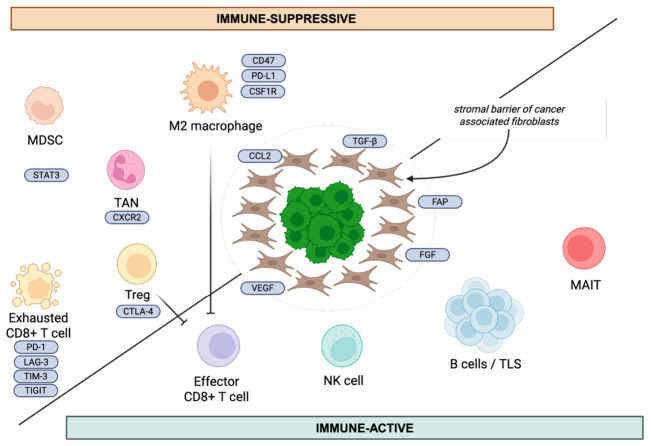
The immunological landscape of cholangiocarcinoma. Schematic representation of the cholangiocarcinoma (CCA) tumor microenvironment, organized along a spectrum from an immune-suppressive (top) to an immune-active (bottom) state. *Abbreviations: CCL2, C-C motif chemokine ligand 2; CD47, cluster of differentiation 47; CSF1R, colony-stimulating factor 1 receptor; CTLA-4, cytotoxic T-lymphocyte–associated protein 4; CXCR2, C-X-C motif chemokine receptor 2; FAP, fibroblast activation protein; FGF, fibroblast growth factor; LAG-3, lymphocyte-activation gene 3; MAIT, mucosal-associated invariant T cell; MDSC, myeloid-derived suppressor cell; NK, natural killer cell; PD-1, programmed cell death protein 1; PD-L1, programmed death-ligand 1; SIRPα, signal regulatory protein alpha; STAT3, signal transducer and activator of transcription 3; TAM, tumor-associated macrophage; TAN, tumor-associated neutrophil; TGF-β, transforming growth factor beta; TIGIT, T cell immunoreceptor with Ig and ITIM domains; TIM-3, T cell immunoglobulin and mucin-domain containing-3; TLS, tertiary lymphoid structure; Treg, regulatory T cell; VEGF, vascular endothelial growth factor.* Figure *created on biorender.com*.

Collectively, these studies establish that CCA immune evasion is orchestrated through multiple parallel and partially overlapping axes: a lymphoid axis driven by Treg-mediated CD8+ suppression, a myeloid axis driven by TAM and MDSC co-dominance, and B cell/TLS axis (Figure 1). This redundancy may in part explain the limited efficacy of single-agent checkpoint blockade in CCA and argues strongly for combination strategies targeting both compartments simultaneously.

### 2.8. Cancer-Associated Fibroblasts in Antitumor Immunity

CCA is among the most desmoplastic of all malignancies, and cancer-associated fibroblasts are the numerically dominant stromal cell type in CCA, representing a critical driver of direct tumor promotion, physical barrier, and immune exclusion. Comprehensive scRNA-seq characterization of CAF heterogeneity in human iCCA identified six transcriptionally distinct subpopulations, of which CD146+ vascular CAFs (vCAFs) were the most abundant, localized in the tumor core, and uniquely positioned to sustain tumor-promoting IL-6/IL-6R signaling that drives EZH2-mediated epigenetic reprogramming of malignant cells [44]. A second study complemented this by demonstrating through genetic lineage tracing and scRNA-seq that hepatic stellate cells are the primary CAF source in iCCA, further resolving CAFs into myofibroblastic CAFs (myCAFs)—which promote tumor growth via hyaluronan synthase 2—and inflammatory CAFs (iCAFs)—which fuel tumor proliferation via HGF–MET signaling [72]. Beyond direct tumor promotion, CAFs also actively orchestrate immune evasion through both myeloid and lymphoid mechanisms. FAP+ CAFs in iCCA are the primary source of CCL2, driving MDSC recruitment via STAT3 activation; FAP knockdown significantly impaired MDSC infiltration and tumor growth, an effect fully rescued by exogenous CCL2, mechanistically linking CAF identity to myeloid immunosuppression [53]. An additional mechanism of stromal niche formation involves TWEAK/Fn14 signaling, which promotes CAF proliferation and immunosuppressive macrophage recruitment in CCA, with inhibition of this pathway shown to restrict tumor growth in preclinical models [73]. Clinically, the prognostic significance of CAFs in CCA has been demonstrated across multiple independent cohorts: high intratumoral FAP expression is independently associated with worse overall and recurrence-free survival in resected iCCA, while high α-SMA expression in CAFs correlates significantly with larger tumor size and shorter survival [74,75]. Indiscriminate CAF depletion has repeatedly failed in desmoplastic cancers—and may paradoxically accelerate progression by removing tumor-restraining CAF subsets—underscoring the need for subtype-selective therapeutic strategies rather than global CAF ablation [72,76]. Therapeutic targeting of CAFs includes depleting CAF subpopulations, inhibiting CAF activation pathways (such as TGF-β or hedgehog signaling), normalizing the extracellular matrix through enzymatic degradation, or reprogramming CAFs toward a less immunosuppressive phenotype.

## 3. Clinical Evidence for Immune Checkpoint Inhibitors in Cholangiocarcinoma

Immune checkpoint blockade in combination has become an established component of cholangiocarcinoma (CCA) management, driven by positive phase III studies in combination with chemotherapy in advanced disease and as monotherapy in biomarker-directed indications in selected patient populations. Current research is focused on enhancing the efficacy of immunotherapy through novel combination strategies with antiangiogenic agents and tyrosine kinase inhibitors, extending its use into earlier-stage disease. To date, the most promising clinical activity has been observed with the lenvatinib-based GOLP regimen in both advanced and neoadjuvant settings, although these findings remain largely limited to Chinese cohorts and require international validation. The sections below summarize the major completed and ongoing studies in these areas (Table 1).

### 3.1. Immune Checkpoint Inhibitors with Chemotherapy (Chemoimmunotherapy)

The landmark phase III TOPAZ-1 trial, conducted across 17 countries spanning Asia, Europe, and America, randomized 685 patients with previously untreated, unresectable, or metastatic biliary tract cancer (BTC)—including intrahepatic and extrahepatic cholangiocarcinoma and gallbladder cancer—to durvalumab or placebo in combination with gemcitabine and cisplatin [11]. Durvalumab significantly improved median overall survival (OS, 12.8 vs. 11.5 months, *p* < 0.05), progression free survival (PFS, 7.2 vs. 5.7 months, *p* < 0.05), and investigator-assessed objective response rates (ORR, 26.7% vs. 18.7%, *p* < 0.05). Updated analysis after 2 and 3 years of randomization confirmed doubling of long-term survival benefit, with two-year OS of 24.9% versus 10.4% and three-year OS of 14.6% versus 6.9% in favor of durvalumab [13,14]. Importantly, subgroup analyses from TOPAZ-1 suggested consistent benefit across most patient subgroups, including different anatomical subtypes, although the magnitude of benefit varied. Prolonged survival benefit was conserved across clinically relevant subgroups, with no specific subgroup driving long-term survival outcome. The results of TOPAZ-1 were independently confirmed by similar findings in the phase 3 KEYNOTE-966 trial, which assessed pembrolizumab plus chemotherapy in patients with previously untreated, advanced BTC. The trial showed improved OS in the pembrolizumab plus chemotherapy group but did not show statistical differences in PFS and ORR [12]. The key difference between TOPAZ-1 and KEYNOTE-966 is the ability to continue chemotherapy after 8 cycles. Based on these results, the FDA granted approval for durvalumab and pembrolizumab in combination with gemcitabine and cisplatin for advanced biliary tract cancer, establishing a new standard of care [77]. Nivolumab, a PD-1 inhibitor, has also been assessed in combination to gemcitabine and cisplatin in two phase II studies and demonstrated favorable ORR, PFS, and OS in the chemotherapy-naïve population, similar to TOPAZ-1 and KEYNOTE-966, indicating that this is likely a class effect [78,79].

TOPAZ-1 findings have now been confirmed with real-world outcomes. A 2025 study by Rimini et al., building on a prior cohort, examined outcomes for 1358 patients with advanced BTC who received the combination of durvalumab plus cisplatin and gemcitabine [80,81]. The study found that response to treatment was consistent with that in the TOPAZ-1 trial. Many additional studies from around the world have similarly confirmed the real-world safety and effectiveness of the combination regimen reported in TOPAZ-1 [82,83,84].

### 3.2. Immune Checkpoint Inhibitors Monotherapy

A particularly compelling rationale for biomarker-directed immunotherapy in CCA derives from the activity of PD-1 blockade in tumors harboring microsatellite instability-high (MSI-H) or mismatch repair-deficient (dMMR) status. Although MSI-H/dMMR is rare in CCA—occurring in fewer than 3% of cases in most series—it confers markedly enhanced immunogenicity through the accumulation of frameshift-derived neoantigens, rendering affected tumors highly responsive to checkpoint inhibition. In the phase II KEYNOTE-158 basket trial, pembrolizumab monotherapy in patients with previously treated MSI-H/dMMR solid tumors achieved an ORR of 40.9% among 22 patients with cholangiocarcinoma or biliary tract cancer, with a median duration of response of 30.6 months, median OS of 19.4 months, and a 3-year OS rate of approximately 30%—outcomes that stand in striking contrast to the modest benefits observed in biomarker-unselected populations [85]. These results were consistent across the broader cohort of MSI-H/dMMR solid tumors, forming the basis for tissue-agnostic FDA approval of pembrolizumab in this molecular subgroup. Collectively, these data underscore the importance of prospective MSI/MMR testing in all patients with advanced CCA and more broadly illustrate the principle that molecular biomarker selection, rather than tumor histology alone, should guide immunotherapy decision-making in this disease.

Conversely, in unselected populations, checkpoint inhibitor monotherapy yielded disappointing results. Analysis of patients with advanced CCA who had progressed on prior chemotherapy, irrespective of MSI or PD-L1 status, and were treated with pembrolizumab monotherapy in the KEYNOTE-158 trial demonstrated an ORR of 5.8% with a median OS of 7.4 months [86,87]. Median progression-free survival (PFS) was 2.0 months. While the overall response rate was modest, responders exhibited durable responses, with all responses lasting 6 months or greater [87]. The phase Ib KEYNOTE-028 trial assessed the safety and antitumor activity of pembrolizumab monotherapy in 24 patients with previously treated advanced CCA and PD-L1 positive tumors, with positivity defined as membranous PD-L1 expression in ≥1% of tumors and associated inflammatory cells or positive staining in stroma. Objective response rate (ORR) was 13.0%, with a median PFS of 1.8 months and median OS of 5.7 months [87]. Other PD-1 inhibitors including nivolumab and tislelizumab have also been evaluated in CCA with similar modest efficacy in unselected populations. Nivolumab monotherapy demonstrated an ORR of 10–22% in small phase II studies, with higher response rates observed in patients with high PD-L1 expression [88].

Taken together, patients with MSI-H/dMMR tumors can derive an exceptional response to PD-1 inhibition, while the remainder of patients with advanced BTCs have insufficient response to checkpoint inhibitor monotherapy.

### 3.3. Dual Checkpoint Blockade

Combination therapy with anti-PD-1/PD-L1 and anti-CTLA-4 antibodies, which has demonstrated synergistic efficacy in other tumor types such as melanoma and renal cell carcinoma, has been explored in CCA [89,90,91]. The Australian phase II study CA209-538 examined the combination of nivolumab plus ipilimumab in patients with rare cancers, among which were advanced biliary tract cancers (BTCs). Subsequent subgroup analyses found an ORR of 23% among patients with advanced BTC treated with ipilimumab and nivolumab. Median PFS was 2.9 months, and median OS was 5.7 months [92], suggesting a favorable response to combination treatment in the refractory setting. Similar studies have also examined combination durvalumab (anti-PD-L1) and tremelimumab (anti-CTLA-4) in BTC, finding the combination to be modestly effective and with a manageable toxicity profile [93,94]. The randomized phase II CheckMate 848 trial lent broader support, showing higher responses with nivolumab plus ipilimumab than with monotherapy in TMB-high solid tumors (tissue-defined ORR 38.6% vs. 29.8%), albeit in a pan-tumor population that did not specifically include CCA [95]. Together with the activity seen in refractory BTC, these data underpin the inclusion of ipilimumab plus nivolumab as a subsequent-line option for advanced CCA with TMB-high tumors in the 2025 NCCN guidelines [96].

Importantly, while the dual checkpoint blockade approach does generally show improved response rates compared to single-agent PD-1 inhibition, the benefit must be weighed against known increased risk of immune-related adverse events, which has been described across multiple types of cancer [89,97,98,99]. Optimal dosing regimens and patient selection strategies are under investigation to maximize efficacy while minimizing toxicity.

### 3.4. Quadruplet Combination with TKIs

The modest but meaningful survival gains established by TOPAZ-1 and KEYNOTE-966 have prompted investigation of more intensive combination strategies, adding antiangiogenic agents, with multi-targeted receptor tyrosine kinase inhibitors (TKIs), vascular endothelial growth factor inhibitors (VEGFi), or CTLA-4 inhibitors to the PD-1/PD-L1 plus chemotherapy backbone. These approaches are grounded in the mechanistic premise that VEGF signaling, tyrosine kinase receptor activity, and co-inhibitory immune checkpoints each contribute orthogonal axes of immunosuppression within the CCA tumor microenvironment.

Early efforts to target these pathways with dual therapy—combining multi-kinase inhibitors such as lenvatinib with checkpoint inhibitors—have demonstrated encouraging but ultimately insufficient activity. In the phase II LEAP-005 study, lenvatinib plus pembrolizumab in previously treated BTC achieved a median OS of 8.6 months and PFS of 6.1 months, with subsequent updates showing an ORR of 17.6%, median OS of 7.9 months, and PFS of 4.1 months [100,101]. Although these results suggested clinical activity and led to an initial NCCN recommendation in the refractory setting, the benefit proved insufficient and the recommendation was later withdrawn [102]. Together, these data suggest that dual-pathway inhibition alone is unlikely to overcome the complexity of immune resistance, reinforcing the need for more intensive, chemotherapy-containing regimens.

The most compelling evidence has emerged with the GOLP regimen in the neoadjuvant setting—a pattern consistent with the broader oncological observation that regimens with high response rates exert their greatest impact earlier in the disease course where the tumor microenvironment is less immunologically exhausted [103]. The GOLP regimen utilized a quadruplet combination of **G**emcitabine (1000 mg/m^2^) on day 1 and 8, **O**xaliplatin (85 mg/m^2^) on day 1, with the TKIs **L**envatinib (8 mg orally, once daily) and anti-**P**D-1 antibody tislelizumab 300 mg or toripalimab 240 mg on day 1. This regimen has been tested in 4 published clinical trials in China [103,104,105,106]. The initial single-arm phase 2 trial of toripalimab plus lenvatinib and GEMOX in 30 patients with advanced intrahepatic CCA demonstrated a striking ORR of 80%, a median OS of 22.5 months, and a median PFS of 10.2 months, substantially exceeding the efficacy of doublet combinations or standard chemoimmunotherapy in this population [106]. Building on this signal, the ZSAB-TransGOLP study evaluated the GOLP regimen with tislelizumab as a conversion strategy in 41 patients with unresectable locally advanced biliary tract cancer—predominantly iCCA—enrolled across two high-volume hepatobiliary centers in China [104]. The trial met its primary endpoint, achieving an R0 resection rate of 63%, with 68% of patients ultimately undergoing surgery. Median OS in the full analysis set was 30.8 months, and among surgical patients was significantly longer than in those who did not proceed to surgery (30.8 versus 13.4 months; *p* < 0.001), underscoring the impact of conversion surgery as a therapeutic goal [104].

These findings informed the design of the phase 2–3 ZSAB-neoGOLP trial, which randomized 178 patients with resectable but high-risk iCCA—defined by at least one of tumor diameter greater than 5 cm, vascular invasion, multifocal disease, hepatic portal lymph-node metastasis, or elevated CA 19-9 > 37 U/mL—at 11 hospitals in China to either three cycles of neoadjuvant GOLP with tislelizumab followed by surgery or upfront surgery alone, with adjuvant capecitabine (2500 mg/m^2^ per day on days 1–14 of a 21-day cycle for eight cycles) administered to both groups postoperatively. Treatment adherence was high: 94% of patients in the neoadjuvant group completed all planned cycles, and rates of surgical resection (97% vs. 99%) and R0 resection (95% vs. 93%) were similar between neoadjuvant and surgery alone groups. At a pre-specified interim analysis with a median follow-up of 16.9 months, event-free survival was significantly longer in the neoadjuvant group (18.0 versus 8.7 months; *p* < 0.001), crossing the pre-specified efficacy boundary. Radiographic response was achieved in 55% of patients in the neoadjuvant group, with 19% demonstrating major pathological response and 5% achieving pathological complete response in resected specimens. Although OS appeared to favor the neoadjuvant group (median not reached versus 31.4 months; HR 0.43; 95% CI 0.23–0.79; *p* = 0.005), this did not meet the pre-specified significance threshold of 0.0019, as adjusted by the Lan–DeMets O’Brien–Fleming spending function, and data maturity remains insufficient for definitive conclusions. The final OS analysis, planned at 60% data maturity, is eagerly anticipated. Collectively, these data provide compelling evidence that lenvatinib-containing quadruplet therapy confers clinically meaningful benefit in the neoadjuvant setting for intrahepatic CCA and raises the important hypothesis that TKI-mediated vascular normalization and immune modulation may be especially relevant in iCCA.

In sum, integrating lenvatinib-containing quadruplet regimens reflects a growing consensus that overcoming immune resistance in CCA requires simultaneous targeting of angiogenic, kinase, and checkpoint axes alongside chemotherapy. The GOLP conversion and neoadjuvant data offer the strongest signal yet that such intensification benefits intrahepatic disease, though whether these gains extend beyond Chinese cohorts and yield durable survival benefit awaits mature data and Western validation before this can become a new standard of care.

### 3.5. Quadruplet Combination with CTLA-4 Inhibitors

The addition of the CTLA-4 inhibitor tremelimumab to durvalumab plus gemcitabine and cisplatin was explored in a Korean phase II study enrolling 77 patients with advanced BTC, in which tremelimumab was administered at varying schedules alongside the standard chemoimmunotherapy doublet. No incremental benefit was observed with the addition of tremelimumab: PFS (12.3 versus 11.8 months) and OS (18.7 versus 20.2 months) were numerically similar or marginally inferior in the tremelimumab-containing arm compared with durvalumab plus chemotherapy alone, with no apparent improvement in response rate or duration of response [107]. This contrasts with the demonstrated additive activity of tremelimumab combined with durvalumab in hepatocellular carcinoma and raises the possibility that dual checkpoint blockade may preferentially benefit tumor types or microenvironmental contexts characterized by higher baseline immune infiltration—a hypothesis particularly relevant to combined HCC-CCA, where CTLA-4 inhibition may yet prove active. Whether intrahepatic CCA, which harbors a more immune-permissive microenvironment, or whether specific genomic subtype, such as IDH1 mutated tumors, may respond differently to dual checkpoint blockade remains an important unanswered question. For now, the addition of CTLA-4 blockade to chemoimmunotherapy is not used routinely in CCA and remains investigational pending data that may identify the subtypes most likely to benefit.

### 3.6. Quadruplet Combination with VEGF Inhibitors

Anti-VEGF strategies, informed by established activity in non-small cell lung cancer and cervical cancer, were evaluated in the global, randomized, double-blind, proof-of-concept phase II IMbrave151 trial, which assigned 162 patients with previously untreated advanced BTC 1:1 to atezolizumab (1200 mg) plus bevacizumab (15 mg/kg) or placebo, both combined with gemcitabine and cisplatin [108]. The addition of bevacizumab yielded a modest but consistent improvement in PFS (8.3 versus 7.9 months; HR 0.67; 95% CI 0.46–0.95) and significantly prolonged median duration of confirmed response (10.3 versus 6.2 months), with 12-month ongoing response rates of 47.8% versus 9.6%. However, ORR was identical between arms (26.6% versus 26.5%), and OS did not differ (14.9 versus 14.6 months), precluding a practice-changing conclusion [108]. Notably, exploratory transcriptomic analysis in 95 biomarker-evaluable patients identified high *VEGFA* gene expression as a candidate predictive biomarker of PFS benefit in the bevacizumab arm (HR 0.44; 95% CI 0.23–0.83), while the broader angiogenesis gene signature did not differentiate benefit [108]. Intriguingly, *VEGFA* expression was highest in intrahepatic CCA and gallbladder cancer relative to extrahepatic CCA, and deconvolution analysis identified hepatocyte enrichment as an additional correlate of bevacizumab benefit, consistent with the hepatic cellular context of intrahepatic CCA [108]. Taken together with the established efficacy of bevacizumab combined with atezolizumab in hepatocellular carcinoma and the pronounced activity of the GOLP regimen in intrahepatic CCA, these observations converge on the hypothesis that angiogenesis is a particularly tractable therapeutic target in intrahepatic rather than extrahepatic biliary malignancies—a distinction with important implications for future biomarker-stratified trial design. At present, the addition of bevacizumab to chemoimmunotherapy is not routinely used in CCA and remains investigational.

### 3.7. Bispecific Antibody

Beyond chemoimmunotherapy doublets, the next frontier involves bispecific antibody constructs designed to simultaneously engage multiple immune checkpoints. GEMINI-Hepatobiliary substudy 2 is a phase II clinical trial testing the use of CTLA-4/anti-PD-1 bispecific antibodies (volrustomig) or anti-PD-1/TIGIT bispecific antibodies (rilvegostomig) in combination with chemotherapy (gemcitabine and cisplatin) in patients with locally advanced or metastatic BTC [109]. The preliminary results of 30 patients treated with rilvegostomig showed an efficacy signal with OOR of 31% and median PFS of 8.3 months [110]. ARTEMIDE-Biliary 02 is also underway and investigating efficacy of rilvegostomig in combination with chemotherapy compared to durvalumab with chemotherapy in BTC [111]. Given the high recurrence rates following CCA resection, adjuvant immunotherapy represents an important unmet need, and the role of rilvegostomig is also being explored in the ARTEMIDE-Biliary01—a global phase III randomized placebo controlled study in combination with capecitabine or gemcitabine/cisplatin in BTC. This trial has completed accrual, and we eagerly await the results. Initial studies evaluating the use of adjuvant immunotherapy in resected CCA have shown promising survival activity [112,113]. Several ongoing clinical trials are currently underway, with eagerly anticipated results [114,115]. Importantly, selection of appropriate patient populations, treatment duration, and biomarker-guided approaches will be key considerations for adjuvant therapy development. With the emergence of PD1-VEGF bispecific antibodies such as ivonescimab and pumitamig, the benefit of targeting VEGF in CCA also remains a possibility. Collectively, these bispecific and PD-1/VEGF approaches span early- and late-phase development but all remain investigational and do not yet impact routine clinical practice.

### 3.8. Immunotherapy and Targeted Therapy

The molecular heterogeneity of CCA, with actionable alterations in FGFR2, IDH1, BRAF, HER2, and other genes in 20–30% of patients, has prompted investigation of immunotherapy combined with molecularly targeted agents. The rationale for such combinations includes potential synergistic antitumor effects and modification of the immunosuppressive tumor microenvironment by targeted agents.

Preclinical studies across tumor models show that FGFR inhibition reshapes the immunosuppressive microenvironment through multiple mechanisms: boosting T cell activation and recruitment, reducing MDSCs and Tregs, reversing M2 macrophage polarization, inactivating CAFs to relieve T cell exclusion, and increasing tumor MHC I/II expression via MAPK/ERK blockade; however, FGFR2:BICC1 fusion junctions themselves can generate neopeptides capable of eliciting neoantigen-specific T cell responses [22,36,116,117]. These findings provide a strong rationale for combination with immune checkpoint inhibitors. Early clinical data in CCA are consistent with this rationale but remain limited. In the combination dose-finding and dose-expansion cohorts of FIGHT-101, pemigatinib plus pembrolizumab produced an objective response rate (ORR) of 26.9% in 26 patients with FGF/FGFR-altered advanced malignancies, with pharmacokinetic and safety profiles consistent with monotherapy exposure [118]. Case reports in advanced iCCA with *FGFR2::BICC1* fusions have documented durable first-line responses to pemigatinib combined with pembrolizumab and platinum-based chemotherapy [119]. Reflecting the strength of the mechanistic rationale, an ongoing phase I/II study (NCT06439485) is evaluating the triplet of pemigatinib, atezolizumab, and bevacizumab in pretreated advanced CCA with FGFR2 fusions or rearrangements [120]. In parallel, a separate phase II study (NCT05174650) is evaluating the combination of derazantinib, an FGFR1-3, CSF1R, and VEGFR2 inhibitor, with atezolizumab in advanced iCCA with FGFR2 fusions or rearrangements [36]. Further combination trials are underway in China and Europe evaluating pemigatinib with the PD-1 inhibitor sintilimab (NCT05913661) and with durvalumab (NCT06530823) in BTC [121,122]. Whether the striking preclinical synergy and early-phase results translate into clinical benefit and eventual integration of FGFR inhibitor plus immune checkpoint inhibitor combination routine practice will depend on the results of these ongoing biomarker-stratified studies.

Combinations of IDH1 inhibitors with immune checkpoint inhibitors in CCA are supported by a strong mechanistic rationale as previously described, although clinical evidence to date has been mixed. In the phase II basket trial (NCT04056910), ivosidenib plus nivolumab yielded limited activity (ORR 6.7%, median PFS 1.94 months) in a heavily pretreated cohort, with all iCCA patients achieving stable disease but no objective responses [123]. While pharmacodynamic effects—including reduction in plasma 2-HG and evidence of immune modulation—were observed, efficacy was comparable to IDH1 inhibition alone, suggesting that PD-1 blockade may not optimally align with the underlying biology. This has prompted ongoing evaluation of alternative combinations in CCA, including triplet regimens incorporating CTLA-4 inhibition (NCT05921760), which more closely mirror preclinical synergy [124]. Moving forward, biomarker-driven trials with paired tissue analyses will be critical to confirm target engagement (e.g., cGAS–STING reactivation, ERV induction) and to identify the subset of patients in whom this strategy translates into meaningful clinical benefit. While the combinations under investigation remain early-phase and have not yet entered routine practice, they represent a promising and biologically rational strategy with the potential to reshape the treatment landscape.

### 3.9. Radiation Therapy with Chemoimmunotherapy

Radiation therapy has well-documented immunomodulatory effects including upregulation of MHC molecules and checkpoint ligands, alteration of the cytokine milieu, and generation of abscopal effects whereby treatment of one tumor site produces immune-mediated regression of distant, unirradiated tumors [125,126,127]. Combination of radiation with immune checkpoint inhibitors have been postulated to enhance this effect but the optimal radiation dose, fractionation, and sequencing with immunotherapy are the subject of further investigation.

Previous studies combining chemotherapy with locoregional therapies in the oligo-metastatic setting established the safety of this approach but yielded mixed efficacy results. The MISPHEC trial was a phase II single arm study, evaluating the combination of yttrium-90 (Y90) selective internal radiation therapy (SIRT) concurrent with cisplatin/gemcitabine in unresectable intrahepatic cholangiocarcinoma and reported encouraging outcome with a median OS of 22 months and median PFS of 14 months [128]. This outcome is superior to mOS and mPFS from patients who received chemotherapy alone from a historic clinical trial such as the ABC-02. However, ABC-07, a randomized phase II trial in which patients with locally advanced biliary tract cancer who had not progressed after induction cisplatin/gemcitabine were randomized to consolidative stereotactic body radiotherapy (SBRT) or continued chemotherapy alone, did not show an improvement in progression free survival [128]. Several design differences likely account for these discordant results. SIRT delivers a high, ablative intratumoral dose transarterially to liver-confined disease—capable of downstaging to resection—whereas SBRT delivers a lower, focal dose limited by adjacent normal-tissue tolerance. The two trials also differed in timing and population: MISPHEC gave SIRT upfront with first-line chemotherapy in intrahepatic-only disease, whereas ABC-07 delivered SBRT only as consolidation in non-progressors and enrolled broader locally advanced BTC, including extrahepatic subtypes in which extrahepatic progression may outpace any local-control benefit.

These insights are now informing the design of ongoing trials that incorporate immunotherapy into this approach, including a single-arm phase II trial (NCT05655949) assessing the safety and efficacy of Y90—administered early in the treatment course—combined with durvalumab, gemcitabine, and cisplatin in biliary tract cancer with liver involvement, with eagerly anticipated results [129]. Extrapolating the data from these studies, many institutions are actively utilize the combination of radiation therapy with chemoimmunotherapy particularly in patients with unresectable BTCs with liver only disease or in the oligometastatic setting.

## 4. Biomarkers for Response Prediction and Resistance Mechanism

Despite promising developments, several challenges complicate the interpretation and implementation of immunotherapy in CCA. Patient heterogeneity, including variations in anatomical subtype, etiology, molecular features, and prior treatments, contributes to inconsistent trial outcomes. Standardization of patient selection criteria and stratification factors in clinical trials is needed to better identify responders.

Biomarker development and validation remain critical unmet needs. While PD-L1 expression, MSI-H/dMMR status, and TMB have shown predictive value in some studies, their utility is limited by methodological variability, incomplete correlation with response, and applicability to only a subset of patients. Development of more robust predictive biomarkers, potentially incorporating multiple parameters including immune profiling, molecular features, and radiographic characteristics, is essential.

### 4.1. Conventional Markers of Response

Biomarker development in CCA has lagged behind the clinical deployment of chemoimmunotherapy, and MSI-high/mismatch-repair-deficient status remains the only tumor-agnostic biomarker with validated predictive value in BTC, supporting pembrolizumab monotherapy for the ~2% of patients with these alterations [130]. In contrast, PD-L1 immunohistochemistry has performed inconsistently. Both TOPAZ-1 and KEYNOTE-966 demonstrated that durvalumab and pembrolizumab provide comparable survival benefit irrespective of PD-L1 expression levels, but interpretation is further confounded by marked variability among antibody clones (22C3, 28-8, SP142, SP263), differing scoring frameworks (TPS vs. CPS), and the absence of a CCA-specific cutoff [131]. Tumor mutational burden shows modest enrichment of responders in pan-tumor analyses, but the generally low-to-intermediate TMB of CCA, non-standardized assay platforms, and lack of a validated threshold limit its stand-alone utility; its 2025 NCCN-endorsed role in CCA is restricted to identifying candidates for ipilimumab–nivolumab in TMB-high refractory disease [96,132,133].

### 4.2. Immune Gene Signatures, Immune Profiling, and Genotype

A second generation of biomarker strategies with multiparametric immune profiling of the tumor microenvironment has increasingly refined patient selection for chemoimmunotherapy in CCA. Transcriptomic signatures capturing T cell inflammation, IFN-γ response, and immune-exclusion phenotypes have predicted ICI benefit across tumor types and correlate with TME subtype in CCA [15,19,20,134]. Within this framework, TLSs have emerged as a central determinant of therapeutic sensitivity: intratumoral TLSs—quantified by four gene-expression signatures—are associated with prolonged PFS and OS in a 100-patient CCA cohort receiving adjuvant chemoimmunotherapy, whereas peri-tumoral TLS correlate with inferior outcomes, similar to prior study in the resected context, underscoring the importance of spatial context [135]. Chung and colleagues subsequently confirmed in 16 resection tumor samples from a phase II trial of nivolumab plus modified gemcitabine/S-1 as first line systemic treatment that TLS-positive tumors had significantly higher ORR, DCR, PFS, and OS than TLS-negative tumors and identified distinct T cell transcriptional programs in TILs and TLS that distinguish responders [63]. Ji and colleagues further integrated radiomic and transcriptomic features into a three-gene immune-response signature (*PLAUR*, *CD40LG*, *FGFR4*) that stratified ICI benefit with AUC 0.84 in a CCA validation cohort [136].

A complementary prospective study by Xu and colleagues of 66 advanced iCCA patients receiving GC plus durvalumab supports the relevance of TME remodeling: responders showed 135 differentially expressed genes enriched in immune-regulatory and mesenchymal pathways, and post-treatment biopsies demonstrated increased PD-L1 expression, expanded CD8+ T cell infiltration, γδ T cell involvement, and reduced α-SMA stromal marking, with 15.2% of patients ultimately achieving R0 conversion resection [137]. Tang and colleagues extended the tissue-based immune-checkpoint repertoire beyond PD-L1 by demonstrating that LAG-3 expression by immunohistochemistry in 44 advanced BTC samples from the T1219 trial predicted chemoimmunotherapy response in a dose-dependent manner (ORR 33%, 58%, and 100% for LAG-3 expression of <1%, 1–9%, and ≥10%, respectively; *p* = 0.018) and correlated with PD-L1 positivity and CD8+ T cell enrichment [138]. LAG-3 expression also correlated with PD-L1 positivity and CD8+ T cell enrichment, suggesting its potential role as a more consistent checkpoint biomarker in this setting. Milardi and colleagues extend this framework by linking B cell biology directly to chemoimmunotherapy responsiveness in iCCA [139]. Single-cell profiling showed that intratumoral B cells are sparse, immature, and functionally suppressed by IL-6 and TGF-β-mediated signaling, whereas mature tertiary lymphoid structures in adjacent tissue track with favorable outcomes [139]. Critically, in patients treated with first-line chemoimmunotherapy, circulating BAFFR+ B cells, higher BAFF levels, and early expansion of treatment-emergent B cell clonotypes were enriched in responders and associated with improved PFS and OS, nominating B cell activation state as a dynamic biomarker of therapeutic efficacy [139]. Mechanistically, dual IL-6/TGF-β blockade restored B cell activation and differentiation ex vivo, supporting this axis as both a predictive biomarker and a rational target to enhance chemoimmunotherapy response [139].

Beyond immune composition, integration of clinical features with H&E images with computational approaches further refine prediction. Cao and colleagues reported a pathomics-based machine-learning signature (PS-ICC) derived from H&E digital images of 189 unresectable iCCA patients on chemoimmunotherapy; PS-ICC was strongly associated with OS (training-cohort HR 0.09; validation-cohort HR 0.20), outperformed RECIST 1.1 as a surrogate for 1-year survival (AUC 0.868 vs. 0.701), and low PS-ICC scores correlated with reduced M0 macrophage infiltration and enrichment of PD-L1/PD-1 checkpoint pathways [140].

Multiple genomic and pathway-level analyses also represent an important axis, although results are not uniformly concordant. Pirrone and colleagues showed that HRD/BRCAness-pathway alterations were associated with improved OS (23.3 vs. 13.8 months, HR 0.51) and PFS (13.2 vs. 8.1 months, HR 0.53) and that TGF-β pathway alterations independently predicted longer PFS (16.0 vs. 8.1 months, HR 0.53) [141]. In a parallel multi-omics study of 125 advanced BTC patients receiving first-line chemoimmunotherapy, Ni and colleagues combined targeted DNA sequencing with bulk RNA sequencing to show that mutations in *TP53*, *BRCA2*, and cytokine-pathway genes, together with high TMB, tracked with response, whereas *KRAS* G12D and *ARID1A* mutations portended poor survival. In a smaller multicenter Japanese cohort of 19 unresectable iCCA patients receiving gemcitabine/cisplatin/durvalumab, *TP53* mutation was also identified as the only independent predictor of shorter PFS on multivariate analysis (HR 4.20), with spatial transcriptomic profiling revealing that *TP53*-mutant tumors displayed a distinct immunosuppressive phenotype characterized by poor CD8+ T cell infiltration, enrichment of CD109+ tumor-associated macrophages, and downregulation of the antigen-presentation genes *TAP1* and *TAP2* [142]. These findings are further contextualized by transcriptomic TME classifications (Job, Martin-Serrano, Carapeto, and Bao for iCCA; Montal for eCCA, defining an “Immune” class in ~11.5% of tumors) and by driver-stratified approaches suggesting that *FGFR2*- and *IDH1*-altered tumors tend toward immunologically cold phenotypes while *ERBB2*-, *KRAS*-, and *TP53*-altered tumors have more immune infiltration in the microenvironments as previously described in the review. Collectively, these data underscore that genomic correlates of chemoimmunotherapy response in BTC are complex, context-dependent, and not uniformly concordant across studies. Importantly, most available datasets remain limited by modest sample sizes, heterogeneity in disease subtype and treatment exposure, and variable depth of molecular profiling, which constrain the stability and generalizability of individual biomarker associations. As such, the effect of specific alterations—particularly targetable genomic events—should not be interpreted in isolation but rather within their broader genomic and tumor microenvironment context. These nuances have direct implications for trial design: rational combination strategies will require mutation-specific frameworks that account for co-alterations, immune phenotype, and functional biology rather than assuming uniform behavior across genomic subgroups.

### 4.3. Circulating Biomarkers

Given the heterogeneity of CCA and the invasive nature of repeat biopsy, dynamic circulating and peripheral biomarkers offer a third, complementary layer. Liquid biopsies, including assessment of circulating tumor DNA (ctDNA), circulating tumor cells (CTCs), and immune-related peripheral blood markers offer potential advantages in accessibility, serial monitoring, and capturing tumor heterogeneity. Plasma ctDNA monitoring has shown early utility in tracking ICI response in BTC, with early declines in variant allele frequency correlating with radiographic benefit [143]. In addition, a study of patients with resectable extrahepatic CCA who underwent longitudinal ctDNA monitoring found that ctDNA positivity at any time after surgery (either prior to or during adjuvant therapy) was associated with significantly inferior disease-free survival [144]. Immune-related peripheral blood markers, including cytokine profiles, may also be potentially useful biomarkers for CCA given success in other cancer types [145,146]. Peripheral markers of inflammation, such as elevated neutrophil-to-lymphocyte ratio, have already been shown to have prognostic value in cholangiocarcinoma; additional studies are needed to determine if these markers may also be predictive of response to immunotherapy [147,148]. Milardi and colleagues found that elevated frequencies of circulating BAFFR+ B cells and hyperexpanded B cell clonotypes were associated with improved chemoimmunotherapy response, providing a peripheral readout of the tissue-level B cell dysfunction described above [139]. Peripheral immune profiling offers a complementary, readily deployable readout. Keenan and colleagues applied CITE-seq to PBMCs from advanced BTC patients on anti-PD-1 and identified a circulating CD14+ monocyte state (CD14_CTX) that expanded in non-responders; the derived gene signature predicted shorter survival in the TCGA cholangiocarcinoma cohort (21.1 months vs. not reached; *p* = 0.02) [149]. The state is defined by two surface markers, Tim-3 and CD29, assayable by routine clinical flow cytometry—offering a practical path to a blood-based assay for patient selection and on-treatment monitoring. Fan and colleagues evaluated the CALLY index (CRP–albumin–lymphocyte composite) in a 110-patient propensity-score-matched CCA cohort on chemoimmunotherapy, showing that high CALLY (>1.42) was associated with longer median OS (13.0 vs. 11.5 months, HR 0.68) and PFS (7.5 vs. 6.0 months, HR 0.70), with time-dependent AUCs of 0.74–0.90 and slower deterioration of health-related quality of life [150]. Memory T cells have also been shown to act as positive predictive biomarkers for response to immune checkpoint inhibition [151,152].

Taken together, these data suggest that biomarker development for ICI use in CCA is best conceptualized as a layered continuum rather than a single defining feature. Established markers such as MSI/dMMR status and actionable driver alterations provide a useful foundation, while a growing body of work illustrates how tissue-based characteristics—including tumor microenvironment subtypes, spatial immune organization (e.g., TLS and B cell biology), checkpoint expression beyond PD-L1, and pathway-level features such as HRD/BRCAness or TGF-β signaling—can further refine the likelihood of response. Complementary studies also highlight the potential role of dynamic peripheral markers, such as ctDNA kinetics, composite inflammatory indices (e.g., CALLY), and circulating immune cell profiles, in capturing treatment response overtime and may be important for treatment de-escalation. Collectively, these examples underscore that no single biomarker is sufficient in isolation; rather, integrating signals across genomic, tissue, and peripheral compartments will likely be necessary to more accurately guide patient selection. Prospective validation of such integrated approaches within ongoing chemoimmunotherapy and targeted–ICI combination studies will be critical to determine their clinical utility.

## 5. Immunotherapy Resistance Mechanisms and Strategies to Overcome Them

### 5.1. Primary Resistance Mechanisms

Primary resistance to immunotherapy, evidenced by lack of initial response, occurs in the majority of CCA patients treated with checkpoint inhibitors. Multiple mechanisms contribute to primary resistance, including intrinsic features of the tumor and its microenvironment.

Lack of tumor immunogenicity due to low mutational burden and paucity of neoantigens represents a fundamental barrier to effective antitumor immunity in many CCA cases [153]. Tumors with few recognizable neoantigens fail to elicit robust T cell responses even when checkpoint inhibition removes inhibitory signals. Immune exclusion, whereby T cells are present in the tumor periphery but fail to infiltrate the tumor parenchyma, is also frequently observed in CCA [7]. The dense desmoplastic stroma characteristic of CCA creates physical barriers to T cell infiltration. Additionally, aberrant tumor vasculature and lack of appropriate chemokine gradients impair T cell trafficking to tumor sites [154]. Finally, immunosuppressive cellular populations, including Tregs, MDSCs, and M2-polarized TAMs, are abundant in many CCA tumors and actively suppress antitumor immune responses [15,155]. High ratios of immunosuppressive to effector immune cells within the TME are known to predict poor immunotherapy responses.

Major histocompatibility complex (MHC) class I molecules, encoded by human leukocyte antigen (HLA) genes, present intracellular peptides to CD8+ T cells, enabling immune recognition of tumor cells. Loss or downregulation of HLA expression represents a potent mechanism of immune evasion, as defects in antigen presentation machinery render tumor cells invisible to T cells despite checkpoint inhibition. HLA loss has been documented in a subset of CCA cases, occurring through various mechanisms including chromosomal deletions, loss of heterozygosity, transcriptional downregulation, or mutations in genes involved in antigen processing and presentation machinery, and has been found to be associated with a “non-inflamed” tumor microenvironment [156,157]. Defects in antigen presentation cannot be overcome by T cell-mediated checkpoint inhibition alone and may require alternative immunotherapeutic approaches such as NK cell-based therapies or interventions to restore HLA expression [157]. Further studies aimed at understanding the patterns and mechanisms of HLA alterations in CCA will be crucial for predicting immunotherapy resistance and developing complementary therapeutic strategies.

Driver genotype including IDH1, FGFR2, KRAS and 9p21 co-deletion of MTAP/CDKN2A imposes a metabolic immunosuppressive program and provide an additional layer of primary-resistance biology that has been detailed in Section 2. The implication for trial design is that genotype-informed combination strategies—IDH1 or FGFR2 inhibition, MTA-depleting enzymes in MTAP-null tumors with checkpoint blockade or other immunotherapeutics—offer more mechanistically coherent approaches than biomarker-agnostic PD-1 blockade.

### 5.2. Acquired Resistance Mechanisms

Acquired resistance develops in patients who initially respond to immunotherapy but subsequently progress. Understanding mechanisms of acquired resistance is crucial for developing strategies to maintain durable responses.

Loss of neoantigen expression through immunoediting represents a well-described mechanism of acquired resistance. Under selective pressure from antitumor immunity, tumor cells with loss or downregulation of immunogenic neoantigens gain survival advantage and eventually dominate the tumor population [158]. This process can occur through deletion of chromosomal regions encoding neoantigens, transcriptional silencing, or selection of pre-existing subclones lacking the targeted antigens.

Acquired defects in interferon signaling pathways, including mutations in JAK1/JAK2 or alterations of interferon-gamma receptor genes, have been identified in tumors developing resistance to checkpoint inhibitors [159,160]. These defects impair tumor cell responsiveness to interferon-gamma produced by activated T cells, thus preventing downstream induction of antigen presentation machinery and allowing immune evasion.

Upregulation of alternative immune checkpoint molecules represents an adaptive resistance mechanism wherein tumors compensate for blockade of one checkpoint pathway by increasing expression of others [160]. For example, tumors treated with PD-1 inhibitors may upregulate TIM-3, LAG-3, or other checkpoints, suggesting potential benefit from sequential or combinatorial checkpoint blockade targeting multiple pathways [161,162].

Epithelial–mesenchymal transition (EMT) in tumor cells represents another mechanism of acquired resistance to immunotherapies. EMT has been associated with immunotherapy resistance through multiple mechanisms including induction of an immune suppressive tumor microenvironment, increased expression of immunosuppressive ligands such as PD-L1, and altered metabolic properties [160].

Additional studies are needed to explore these mechanisms in the context of CCA to develop strategies to better address acquired resistance to immunotherapy in CCA.

### 5.3. Strategies to Overcome Resistance

Combination approaches targeting multiple resistance mechanisms simultaneously represent the most promising strategy for improving immunotherapy outcomes in CCA. Rational combinations should be determined based on an understanding of the specific mechanisms driving each patient’s resistance.

Combination of checkpoint inhibitors with agents that enhance tumor immunogenicity, such as chemotherapy, radiation therapy, or oncolytic viruses, may convert immunologically cold tumors into hot tumors. These treatments induce immunogenic cell death, releasing tumor antigens and danger signals that activate antitumor immune responses. The success of chemotherapy–immunotherapy combinations in the TOPAZ-1 trial validates this approach [14].

Beyond PD-1/PD-L1 and CTLA-4, numerous additional immune checkpoint and co-stimulatory molecules are being investigated as therapeutic targets. Next-generation checkpoint inhibitors targeting LAG-3, TIM-3, TIGIT, VISTA, B7-H3, and others are in clinical development, either as monotherapy or in combination with PD-1/PD-L1 blockade [163,164,165,166].

Agonistic antibodies targeting co-stimulatory molecules such as OX40, 4-1BB (CD137), GITR, or CD40 represent an alternative approach to enhance antitumor immunity. These agents activate and expand effector T cells rather than simply removing inhibitory signals. Combination of co-stimulatory agonists with checkpoint inhibitors may produce synergistic effects, although careful attention to dosing and sequencing is required to avoid excessive toxicity [167,168].

Bi-specific and multi-specific antibodies that simultaneously engage multiple immune checkpoints or combine checkpoint blockade with co-stimulation represent innovative approaches currently entering clinical evaluation [111,165,169,170,171]. These molecules may offer pharmacological advantages including simplified dosing regimens and enhanced potency compared to combination of separate antibodies.

Targeting the immunosuppressive tumor microenvironment through inhibition of pathways such as TGF-β, adenosine, or IDO can synergize with checkpoint blockade [171,172]. Multiple early-phase clinical trials are evaluating these combinations, with promising preliminary results in some studies [173,174,175]. Targeting the adenosine pathway through inhibition of CD73 or adenosine receptors represents another approach to reverse immunosuppression. CD73 inhibitors are being evaluated in combination with checkpoint inhibitors in multiple solid tumor types including biliary tract cancers.

Modulation of the stromal compartment, particularly targeting CAFs, or modifying the extracellular matrix to enhance immune cell infiltration and drug delivery, represents an important complementary strategy [176]. Preclinical studies have demonstrated that combining CAF-targeting agents with checkpoint inhibitors can overcome resistance and improve outcomes [177,178,179].

## 6. Emerging Immunotherapeutic Strategies

### 6.1. Adoptive Cell Therapy

Adoptive cell therapy (ACT) involves the collection, ex vivo manipulation, and reinfusion of immune cells to mediate antitumor responses. Several ACT approaches are being investigated in CCA, including tumor-infiltrating lymphocyte (TIL) therapy, engineered T cell receptor (TCR) therapy, and chimeric antigen receptor (CAR) T cell therapy. Thus far, these have been mostly investigated in preclinical and case report settings, and extensive clinical trial efforts are ongoing in this space (Table 2).

TIL therapy is based on the premise that tumor-resident T cells include clones capable of recognizing patient-specific neoantigens. The modern framework for TIL therapy in CCA dates to the landmark case report from the NIH in 2014 in which whole-exome sequencing of a metastatic iCCA identified a mutation in *ERBB2IP* (E805G) recognized by tumor-derived CD4+ T-helper-1 cells [180]. Adoptive transfer of approximately 42 billion TIL containing ~25% mutation-specific CD4+ cells produced regression of lung and liver metastases; upon progression, retreatment with a >95% pure population of *ERBB2IP*-reactive CD4+ cells induced a second, durable response for more than 10 years [180]. This case provided the first direct evidence that a CD4+ T cell response against a patient-specific neoantigen could mediate regression of metastatic epithelial cancer and catalyzed subsequent development programs in gastrointestinal tumors. This approach has shown remarkable efficacy in melanoma [181,182]. A dedicated phase II study of autologous TIL and high-dose aldesleukin in locally advanced, recurrent, or metastatic BTC (NCT03801083) [183] is currently enrolling at the University of Pittsburgh, and a parallel phase I/II study of TIL genetically engineered with additional checkpoint modulation in metastatic gastrointestinal epithelial cancers includes BTC patients (NCT04426669) [184].

CAR T cell therapy, which has revolutionized treatment of B cell malignancies and multiple myeloma, faces considerable challenges in solid tumors including CCA owing to the absence of truly tumor-restricted surface antigens, poor T cell trafficking into densely desmoplastic stroma, and the immunosuppressive tumor microenvironment as previously described. Published CCA-specific experience to date comes from three small Chinese studies. Feng and colleagues reported a 52-year-old woman with chemorefractory unresectable CCA who received a sequential cocktail of CART-EGFR and CART-CD133 cells, achieving an 8.5-month partial response after EGFR-targeted infusion and an additional 4.5-month partial response after CD133-targeted infusion [185]. Guo and colleagues subsequently extended this experience in a phase I trial of CART-EGFR in 19 patients with EGFR-positive (>50%) advanced BTC (14 cholangiocarcinoma, 5 gallbladder carcinomas): among 17 evaluable patients, 1 achieved complete response and 10 achieved stable disease, with median progression-free survival of 4 months (range 2.5–22) [186]. Enrichment of central memory T cells in the infused product predicted clinical response, foreshadowing the contemporary emphasis on product quality attributes [186]. Feng and colleagues also reported a phase I trial of CART-HER2 in 11 HER2-positive advanced BTC/pancreatic cancer patients, with 1 partial response (4.5 months) and 5 stable disease, median PFS 4.8 months [187]. Ongoing CAR-T programs relevant to CCA span multiple tumor-associated antigens: mesothelin (conventional CAR-T; NCT06256055 [188]), Killer-cell Immunoglobulin-like Receptor (KIR-CAR); NCT05568680 [189]), CEA (NCT06043466 [190]), CDH17 (NCT06937567 [191]), and MUC1 (NCT03633773 [192], specifically in iCCA). A conceptually distinct approach—chimeric antigen receptor macrophages—is being evaluated in HER2-overexpressing solid tumors including BTC (CT-0508, NCT04660929 [193]), and an autologous central-memory T cell product is under investigation specifically in iCCA (NCT03820310 [194]). Early-generation cytokine-induced killer (CIK) cell therapy for CCA is being explored at Siriraj Hospital in Thailand (NCT01868490 [195]).

TCR-engineered T cells, which combine the antigen specificity of TCRs with the ability to recognize intracellular antigens presented by HLA molecules, represent another promising approach [196]. Identification of tumor-specific neoantigens or cancer-testis antigens in CCA could enable development of neoantigen-specific TCR therapies. Ongoing TCR-T programs relevant to CCA include NCT05349890 [197] in incurable epithelial cancers and NCT03907852 [198] (mesothelin-targeting TCR-fusion construct) with additional neoantigen-directed TCR-T platforms in advanced development.

NK cell therapy represents a complementary ACT platform, leveraging innate cytotoxicity without HLA restriction and with a favorable safety profile relative to T cell products. Leem and colleagues reported a multicenter open-label phase 1/2a trial of allogeneic NK cells combined with pembrolizumab in chemotherapy-refractory BTC, demonstrating feasibility and preliminary activity [199]. Engineered NK products, including CAR-NK and cytokine-armored NK platforms, are in earlier-phase development and may be attractive for desmoplastic tumors such as CCA where T cell trafficking is limited.

### 6.2. Myeloid Targeted Therapies

Pharmacologic targeting of the myeloid axis is now being explored clinically in CCA, predominantly through early-phase combination trials. Derazantinib, a multikinase FGFR/CSF1R/VEGFR2 inhibitor, is under evaluation with atezolizumab in FGFR2-altered iCCA [200], and a HER2-directed chimeric antigen receptor macrophage (CT-0508) is in early testing in HER2-expressing solid tumors including BTC [193]. Because single-axis targeting can provoke compensatory escape and TAMs are the principal source of PD-L1 in CCA, effective regimens will likely need to pair a myeloid-directed agent with checkpoint blockade. For example, the CD40 agonist CDX-1140—building on murine iCCA data showing that CD40-mediated myeloid and dendritic-cell activation enhances response to anti-PD-1—is being evaluated with capecitabine/oxaliplatin and pembrolizumab in a dedicated phase I/II biliary tract cancer trial [201,202]. Agents directed at the remaining nodes—CD47–SIRPα phagocytosis-checkpoint blockade (magrolimab, evorpacept) and CCL2/CCR2 or CXCR2 inhibitors (AZD5069)—have advanced predominantly in other solid tumors [203,204,205]. Although no myeloid-targeted agent has yet entered routine CCA practice, this remains a promising area of investigation with the potential to produce more durable antitumor immune responses.

### 6.3. Cancer Vaccines

Cancer vaccines aim to stimulate antitumor immune responses by presenting tumor antigens—delivered as peptides, nucleic acids, dendritic cells loaded with antigen, or whole tumor-cell preparations—to the immune system. CCA was one of the earliest solid tumors in which therapeutic cancer vaccination was formally tested, and the field has recently been reinvigorated by improvements in neoantigen prediction, adjuvant selection, and the ability to combine vaccination with immune checkpoint blockade. Herein, we summarize the development and ongoing clinical trials of cancer vaccines in CCA in this section and in Table 3.

Early peptide-based programs in BTC focused on shared tumor-associated antigens, particularly Wilms’ tumor 1 (WT1) and mucin-1 (MUC1), both frequently overexpressed in cholangiocarcinoma and gallbladder cancer. Yamamoto and colleagues and Lepisto and colleagues reported early-phase studies of a 100-amino-acid MUC1 peptide administered either with incomplete Freund’s adjuvant or pulsed onto autologous dendritic cells, respectively; both demonstrated safety but only sporadic clinical activity [206,207]. Kobayashi and colleagues subsequently reported a retrospective series of 65 patients with nonresectable, recurrent, or metastatic BTC treated with autologous dendritic cells pulsed with WT1 and/or MUC1 peptides, with a median survival of 18.5 months from diagnosis and 7.2 months from first vaccination and independent prognostic significance of combined chemotherapy, baseline albumin and CRP, and post-vaccination fever—an early indication that host inflammatory context modulates vaccine efficacy [208]. Shimizu and colleagues delivered a complementary adjuvant-setting signal in 36 patients with resected iCCA treated with postoperative autologous tumor-lysate-pulsed DC vaccination combined with ex vivo CD3-activated T cell transfer, reporting median progression-free survival of 18.3 months versus 7.7 months and median overall survival of 31.9 months versus 17.4 months for surgery alone [209]. Aruga and colleagues extended the antigen repertoire beyond WT1/MUC1 by administering a four-peptide vaccine derived from cancer-testis antigens (LY6K, TTK, IMP3, DEPDC1) in 9 patients with chemotherapy-refractory advanced BTC, showing peptide-specific CTL induction and an association of Type-1-dominant host immune setup with improved outcomes [210].

An important recent case report by Maia and colleagues, published in *Journal for ImmunoTherapy of Cancer* in 2025, provides striking follow-up data on a patient with metastatic iCCA who underwent repeated surgery combined with two successive rounds of personalized peptide vaccination and remained tumor-free for more than 8 years at the time of reporting [211,212]. In-depth functional immune analyses demonstrated a dominant CD4+ T cell response against the vaccine antigens with infiltration of the tumor site, immune responses persisting for years after the last vaccine administration, and spontaneous detection of neoantigen-specific CD4+ and CD8+ T cells, all of which the authors argue may have contributed to the exceptional outcome [211,212]. While anecdotal, this case is among the most durable responses reported to any form of immunotherapy in CCA and parallels the Tran *ERBB2IP* TIL case in highlighting the potential of CD4+ neoantigen-directed immunity in this disease.

A contemporary proof-of-concept for off-the-shelf driver-mutation vaccination comes from the KRAS-directed amphiphile vaccine ELI-002, which pairs lipid-conjugated mutant-*KRAS* peptides (G12D and G12R in the 2-peptide formulation) with an amphiphile-modified CpG-7909 adjuvant that co-traffics to draining lymph nodes to enhance T cell priming. In the phase 1 AMPLIFY-201 trial (NCT04853017), 25 minimal-residual-disease-positive patients (20 PDAC and 5 CRC) after locoregional therapy received ELI-002; 84% mounted ex vivo mutant-*KRAS*-specific T cell responses (59% with concurrent CD4+ and CD8+), 84% had tumor biomarker reductions, and 24% achieved complete biomarker clearance [213]. In the final analysis at 19.7 months of follow-up, patients whose T cell induction exceeded a 9.17-fold threshold had median radiographic relapse-free survival not reached versus 3.02 months (HR 0.12; *p* = 0.0002) and median overall survival not reached versus 15.98 months (HR 0.23; *p* = 0.0099), with antigen spreading to non-vaccine neoantigens in 67% of patients [214]. Although AMPLIFY-201 did not enroll CCA, the platform is directly relevant to the 10–25% of biliary tract cancers harboring oncogenic *KRAS*—particularly G12D, the dominant allele in iCCA—and the 7-peptide formulation in the ongoing phase 2 AMPLIFY-7P trial (NCT05726864 [215]) covers the full spectrum of common *KRAS* codon-12/13 mutations seen in BTC.

Current CCA/BTC vaccine programs increasingly target driver mutations and shared tumor antigens alongside or instead of broad tumor-lysate preparations. The mBTCvax program at Johns Hopkins (NCT06564623 [216]) combines a peptide vaccine targeting driver oncogenes with dual checkpoint blockade (durvalumab plus tremelimumab) in BTC and represents the most clinically mature of the next-generation CCA vaccine trials. The Globo H-directed conjugate vaccine OBI-833 with immunostimulant OBI-821 (NCT06490198) is planned as maintenance therapy for Globo H-positive BTC after gemcitabine/cisplatin [217]. The flexibility and rapid-production advantages of mRNA vaccine platforms—now validated clinically in melanoma and pancreatic cancer—are particularly attractive for CCA given the disease’s heterogeneity and the scarcity of broadly shared tumor antigens; preclinical work has begun to integrate CCA antigen identification with transcriptomic immune subtyping to guide patient selection [218].

## 7. Conclusions and Perspectives

CCA has transitioned from chemotherapy alone to a biomarker-informed, chemoimmunotherapy-based standard. Phase III trials (TOPAZ-1, KEYNOTE-966) established gemcitabine/cisplatin plus PD-1/PD-L1 blockade, with durable survival gains confirmed in real-world cohorts, while perioperative strategies (e.g., GOLP; emerging adjuvant data) are extending this paradigm into earlier-stage disease. In parallel, deeper interrogation of the tumor microenvironment has shown that limited response rates are driven by convergent resistance biology—low neoantigen burden, stromal exclusion, and myeloid-dominant immunosuppression, often reinforced by genotype-specific “cold” states—whereas acquired resistance reflects antigen loss, HLA downregulation, and adaptive checkpoint reprogramming.

Despite these advances, major gaps persist. No single biomarker reliably stratifies the large majority of pMMR/MSI-stable patients, and anatomical and genomic subtypes remain only loosely integrated into treatment selection. Conflicting biomarker signals across cohorts underscore the need for paired, longitudinal sampling to resolve context-dependent effects, while the optimal sequencing and integration of immunotherapy with locoregional approaches remains undefined. Addressing these challenges will require a shift in trial design and therapeutic strategy. Prospective studies must embed multi-compartment biomarker collection, integrating tissue, spatial, and peripheral readouts to capture the full complexity of the disease. At the same time, combination strategies should move beyond established targets to include genotype-defined subsets such as KRAS, MTAP, and BRAF, while dynamic monitoring with ctDNA and circulating immune indices offers a path toward real-time treatment adaptation. Finally, prioritizing myeloid and stromal targets—central mediators of immune resistance—may unlock broader responsiveness. Together, these efforts will be essential to transition the field from empiric combinations to mechanism-driven, biomarker-guided therapy. Realizing this shift will be essential to deliver durable, broadly applicable benefit and move CCA toward truly personalized, curative-intent care.

## Figures and Tables

**Table 1:** Key clinical trials of immune checkpoint inhibitor-based regimens in cholangiocarcinoma and biliary tract cancer from approved standards of care to early-phase studies.

Trial/Study	Phase	Setting/Population	Regimen	Key Outcome
**Standard of Care (Approved and Guideline-Recommended)**				
TOPAZ-1	III	Advanced BTC, 1L	Durvalumab + Gem/Cis vs. Gem/Cis	mOS 12.8 vs. 11.5 mo; FDA-approved
KEYNOTE-966	III	Advanced BTC, 1L	Pembrolizumab + Gem/Cis vs. Gem/Cis	mOS 12.7 vs. 10.9 mo; FDA-approved
KEYNOTE-158	II	MSI-H/dMMR BTC/CCA	Pembrolizumab monotherapy	ORR 40.9%; mOS 19.4 mo; tissue-agnostic approval
CA209-538	II	Advanced BTC, refractory	Nivolumab + Ipilimumab	ORR 23%; NCCN-listed for TMB-high (later line)
**Promising Combination**				
ZSAB-neoGOLP	II–III	Resectable high-risk iCCA, neoadjuvant	Tislelizumab + Lenvatinib + GEMOX Vs. Surgery alone	EFS 18.0 vs. 8.7 mo
ZSAB-TransGOLP	II	Unresectable LA BTC conversion	Tislelizumab + Lenvatinib + GEMOX	R0 63%; mOS 30.8 mo
GOLP (initial)	II	Advanced iCCA, 1L	Toripalimab + Lenvatinib + GEMOX	ORR 80%; OS 22.5 mo; PFS 10.2 mo
**Strategies without demonstrated benefit**				
LEAP-005	II	Previously treated BTC	Pembrolizumab + Lenvatinib	ORR 17.6%; OS 7.9 mo; NCCN rec withdrawn
IMbrave151	II	Advanced BTC, 1L	Atezolizumab + Bevacizumab + gem/cis	PFS 8.3 vs. 7.9 mo; OS no difference
NCT03046862	II	Advanced BTC, 1L	Tremelimumab + durvalumab + GemCis vs. durvalumab + GemCis	PFS 12.3 vs. 11.8 mos; OS 18.7 vs. 20.2 mos
NCT04056910	II	IDH1-mutant CCA	Nivolumab +Ivosidenib	ORR 6.7%; PFS 1.94 mo
**Ongoing Studies**				
GEMINI-Hepatobiliary	II	LA/metastatic BTC	Volrustomig or Rilvegostomig + Gem/Cis	Rilvegostomig ORR 31%; PFS 8.3 mo
ARTEMIDE-Biliary 02	III	Advanced BTC, 1L	Rilvegostomig + Gem/Cis vs. Durvalumab + Gem/Cis	Ongoing
ARTEMIDE-Biliary 01	III	BTC, adjuvant	Rilvegostomig + Chemo vs. Chemo	Ongoing
NCT06439485	I/II	FGFR2-altered CCA	Pemigatinib + atezolizumab + bevacizumab	Ongoing
NCT05174650	II	FGFR2-altered iCCA	Derazantinib + atezolizumab	Ongoing
NCT05921760	II	IDH1-mutant CCA	Nivolumab + Ipilimumab + Ivosidenib	Ongoing
NCT05655949	II	BTC	Y90 + durvalumab + gem/cis	Ongoing

*Abbreviations: BTC, biliary tract cancer; DoR, duration of response; EFS, event-free survival; gem/cis, gemcitabine/cisplatin; iCCA, intrahepatic cholangiocarcinoma; LA, locally advanced; 1L, first-line; ORR, objective response rate; OS, overall survival; PFS, progression-free survival; SIRT, selective internal radiation therapy*.

**Table 2:** Selected ongoing adoptive cell therapy trials relevant to cholangiocarcinoma.

Platform	Agent/Target	Phase	Trial	Indication
TIL	Autologous TIL + aldesleukin	II	NCT03801083	Locally advanced, recurrent, or metastatic BTC
TIL (engineered)	TIL with novel checkpoint modulation	I/II	NCT04426669	Metastatic GI epithelial cancers
Central-memory T cells	Autologous Tcm	—	NCT03820310	Intrahepatic cholangiocarcinoma
CIK cells	Modified autologous CIK	—	NCT01868490	Cholangiocarcinoma
CAR-T	MUC1	I/II	NCT03633773	Intrahepatic cholangiocarcinoma
CAR-T	CEA	I	NCT06043466	CEA-positive advanced solid tumors (incl. BTC)
CAR-T	CDH17	I	NCT06937567	CDH17-positive advanced solid tumors
CAR-T	HER2	I	NCT07334119	Advanced HER2-expressing solid tumors
CAR-T	Mesothelin	I	NCT06256055	Mesothelin-positive advanced solid tumors
CAR-T (KIR variant)	Mesothelin KIR-CAR	I	NCT05568680	Ovarian, CCA, or mesothelioma
TCR-fusion	Mesothelin	I/II	NCT03907852	Advanced mesothelin-expressing cancers
TCR-T	Neoantigen/shared antigen	I	NCT05349890	Incurable epithelial cancers
CAR-macrophage	HER2 (CT-0508) ± pembrolizumab	I	NCT04660929	HER2-overexpressing solid tumors (incl. BTC)

*Abbreviations: BTC, biliary tract cancer; CAR, chimeric antigen receptor; CCA, cholangiocarcinoma; CEA, carcinoembryonic antigen; CIK, cytokine-induced killer; GI, gastrointestinal; MUC1, mucin-1; TCR, T cell receptor; Tcm, central-memory T cell; TIL, tumor-infiltrating lymphocyte*.

**Table 3:** Selected ongoing or recently completed cancer vaccine trials relevant to cholangiocarcinoma.

Vaccine Platform	Antigen/Target	Combination	Phase	Trial	Indication
Peptide (mBTCvax)	Driver oncogenes (multi-peptide + poly-ICLC)	Durvalumab + tremelimumab	—	NCT06564623	BTC (Johns Hopkins)
Conjugate (OBI-833 + OBI-821)	Globo H	— (maintenance post-GC)	II	NCT06490198	Globo H-positive BTC
Amphiphile vaccine (ELI-002)	KRAS G12D/G12R and NRAS	—	I/II	NCT04853017 (completed)	KRAS-mutant solid tumors.
Personalized neoantigen DC + precision T cells	Patient-specific neoantigens	Gemcitabine	—	NCT02632019	Advanced BTC

*Abbreviations: BTC, biliary tract cancer; CCA, cholangiocarcinoma; DC, dendritic cell; GC, gemcitabine/cisplatin; poly-ICLC, polyinosinic-polycytidylic acid stabilized with polylysine and carboxymethylcellulose*.

## Data Availability

No new data were created or analyzed in this study. Data sharing is not applicable to this article.
